# Temperature-dependent optical constants of monolayer ${\text {MoS}}_2$, ${\text {MoSe}}_2$, ${\text {WS}}_2$, and ${\text {WSe}}_2$: spectroscopic ellipsometry and first-principles calculations

**DOI:** 10.1038/s41598-020-71808-y

**Published:** 2020-09-17

**Authors:** Hsiang-Lin Liu, Teng Yang, Jyun-Han Chen, Hsiao-Wen Chen, Huaihong Guo, Riichiro Saito, Ming-Yang Li, Lain-Jong Li

**Affiliations:** 1 Department of Physics, National Taiwan Normal University, Taipei, 11677 Taiwan; 2 Shenyang National Laboratory for Materials Science, Institute of Metal Research, Chinese Academy of Sciences, 72 Wenhua Road, Shenyang, 110016 China; 3 Department of Physics, Tohoku University, Sendai, 980-8578 Japan; 4 College of Sciences, Liaoning Shihua University, Fushun, 113001 China; 5 Research Center for Applied Science, Academia Sinica, Taipei, 10617 Taiwan; 6 Physical Science and Engineering Division, King Abdullah University of Science and Technology, Thuwal, 23955-6900 Kingdom of Saudi Arabia

**Keywords:** Materials science, Nanoscience and technology, Optics and photonics, Physics

## Abstract

The temperature-dependent ($T = 4.5 \, \hbox {-} \, 500 \, \hbox {K}$) optical constants of monolayer ${\text {MoS}}_2$, ${\text {MoSe}}_2$, ${\text {WS}}_2$, and ${\text {WSe}}_2$ were investigated through spectroscopic ellipsometry over the spectral range of 0.73–6.42 eV. At room temperature, the spectra of refractive index exhibited several anomalous dispersion features below 800 nm and approached a constant value of 3.5–4.0 in the near-infrared frequency range. With a decrease in temperature, the refractive indices decreased monotonically in the near-infrared region due to the temperature-dependent optical band gap. The thermo-optic coefficients at room temperature had values from $6.1 \times 10^{-5}$ to $2.6 \times 10^{-4} \, \hbox {K}^{-1}$ for monolayer transition metal dichalcogenides at a wavelength of 1200 nm below the optical band gap. The optical band gap increased with a decrease in temperature due to the suppression of electron–phonon interactions. On the basis of first-principles calculations, the observed optical excitations at 4.5 K were appropriately assigned. These results provide basic information for the technological development of monolayer transition metal dichalcogenides-based photonic devices at various temperatures.

## Introduction

Monolayer transition metal dichalcogenides (TMDs) such as ${\text {MoS}}_2$, ${\text {MoSe}}_2$, ${\text {WS}}_2$, and ${\text {WSe}}_2$ have attracted much attention in recent years. This interest has been stimulated not only by their novel physical properties in the reduced dimension^1–5^, but also by their potential applications in nanoelectronic, optoelectronic, spintronic, and valleytronic devices^6–16^. When we fabricate optoelectronic devices for practical applications, systematic study of the optical properties of all TMDs species using the same method is essential. In particular, knowledge about the temperature dependence of optical constants is necessary to understand the effects of self-heating on the performance of devices. The motivation of this work was to investigate the frequency and temperature dependences of monolayer TMDs’ optical constants. Ascertaining these will assist with developing optoelectronic devices in a wide photon energy and temperature range.

Spectroscopic ellipsometry has proved to be an effective tool for non-destructively characterizing the optical properties of monolayer TMDs^17^, providing critical information about the refractive index, extinction coefficient, and thickness of thin films^18^. Using spectroscopic ellipsometry, Yim et al.^19^ measured the optical properties of ${\text {MoS}}_2$ thin films at room temperature for several thicknesses (1.99–19.88 nm). They proposed a model for optical dispersion from which the optical constants and the film thickness values were obtained. They pointed out that thinner ${\text {MoS}}_2$ films have larger band gap energy. Eichfeld et al.^20^ examined the optical properties of ultrathin ${\text {WSe}}_2$ films (2–35 nm) at room temperature using spectroscopic ellipsometry. They stated that the refractive index and extinction coefficient are independent of thickness. In this study, the indirect optical band gap energy of ${\text {WSe}}_2$ was closely correlated to its thickness. Park et al.^21^ presented the temperature dependence of the dielectric function of monolayer ${\text {MoS}}_2$ obtained using spectroscopic ellipsometry. They calculated ten distinct energies in the dielectric function as the critical-point structures, in which the critical-point energy is defined by the second derivatives of the dielectric function. The temperature dependence of the critical-point energies was fitted to a phenomenological expression containing the Bose–Einstein distribution function. Recently, Choi et al.^22^ reported the temperature dependence of band gap in ${\text {MoSe}}_2$ ultrathin films, also through the use of spectroscopic ellipsometry. The band gap decreases linearly with an increase in temperature, which is explained by the vibronic model^23^. Diware et al.^24^ studied the complex dielectric function of monolayer ${\text {WSe}}_2$ using spectroscopic ellipsometry. They found that monolayer ${\text {WSe}}_2$ has an indirect band gap of 2.26 eV and direct band gap of 2.35 eV. Binding energies of A and B excitions were estimated to be 0.71 and 0.28 eV, respectively. Park et al.^25^ measured the temperature dependence of the dielectric function of monolayer ${\text {MoSe}}_2$ using spectroscopic ellipsometry. They observed six critical-point energies at room temperature and six additional structures at 31 K. All structures showed a blueshift and sharpened with a decrease in temperature as a result of the reducing lattice constant and electron–phonon interactions. More recently, Elliott et al.^26^ reported a joint ellipsometric and first-principles characterization of the surface susceptibility and conductivity of monolayer ${\text {MoS}}_2$ and ${\text {WSe}}_2$. They identified the excited exciton peaks in the ellipsometric spectra.

However, most spectroscopic ellipsometry measurements of monolayer TMDs have been limited to a few TMDs (e.g., $${\text {MoS}}_2$$, $${\text {MoSe}}_2$$, and $${\text {WSe}}_2$$) and to narrow frequency or temperature ranges. To obtain systematic knowledge of the frequency and temperature dependences of optical constants ($$ i.e.$$, refractive index and extinction coefficient) of monolayer TMDs, we present a detailed characterization of optical constants in monolayer $${\text {MoS}}_2$$, $${\text {MoSe}}_2$$, $${\text {WS}}_2$$, and $${\text {WSe}}_2$$ over a wide range of photon energy (from 0.73 to 6.42 eV) and temperature (between 4.5 and 500 K). To cooperate with the experimental exploration of temperature and frequency dependences of optical constants, we also present parallel simulations and calculations of the optical properties in monolayer TMDs. From the literature, one can see the complexity of theoretical treatment of the optical properties of TMDs, in terms of spin–orbit coupling, electron–hole interaction induced strong excitonic effects^27–29^. Fortunately, the spin–orbit interaction can be well accounted for by solving the Dirac equation within the relativistic limit^30^. Moreover, excitonic effects can be approximated by scaling up the conduction band states since the density functional theory and quasiparticle band structures are qualitatively similar and mainly differ in band gap energy^31,32^. Thus within the density functional theory and density functional perturbation theory, we can calculate the temperature and frequency dependences of the optical constants which reproduce the experimental results satisfactory.

**Figure 1:**
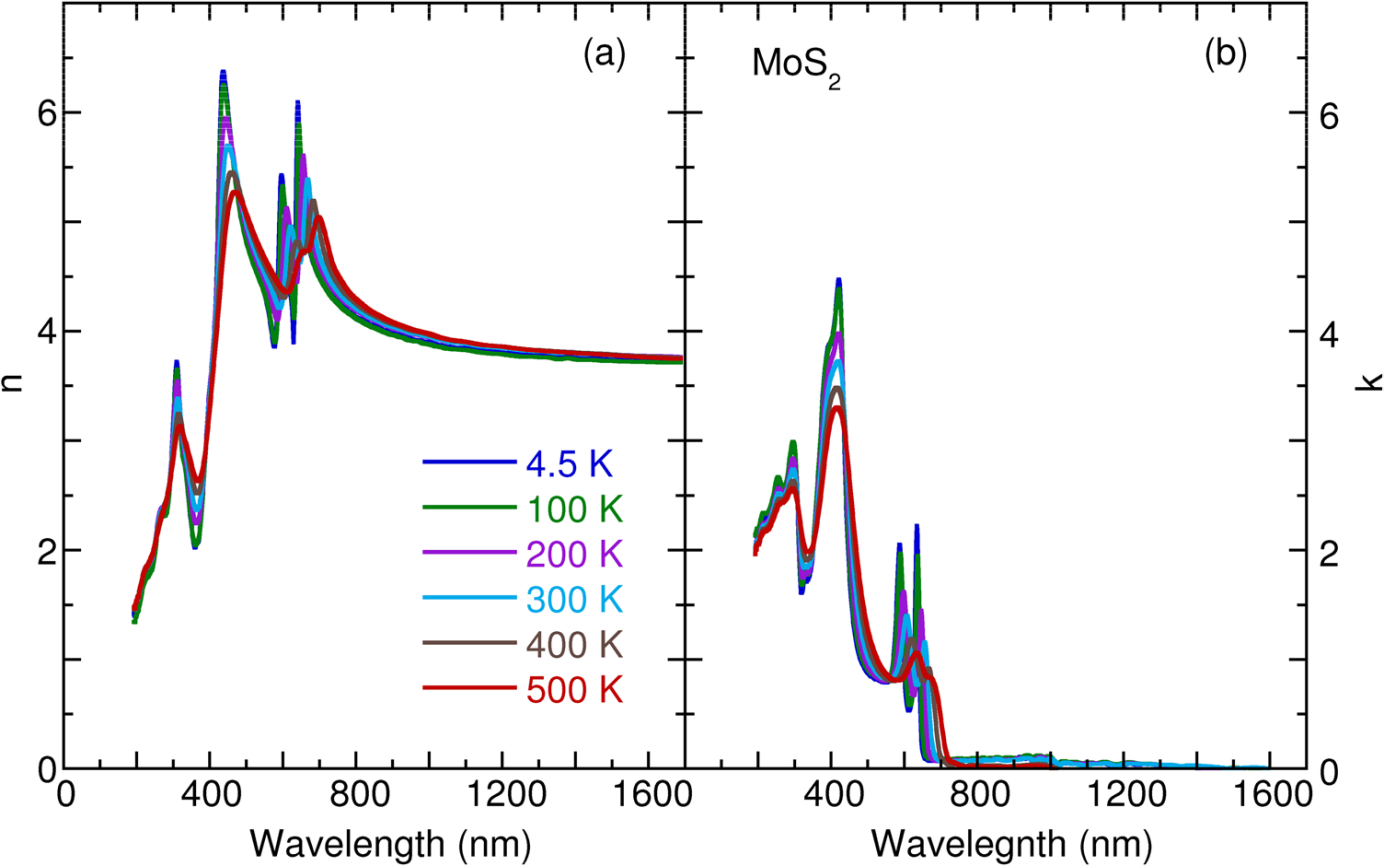
Temperature dependence of (**a**) refractive index *n* and (**b**) extinction coefficient *k* of monolyaer ${\text {MoS}}_2$.

**Figure 2:**
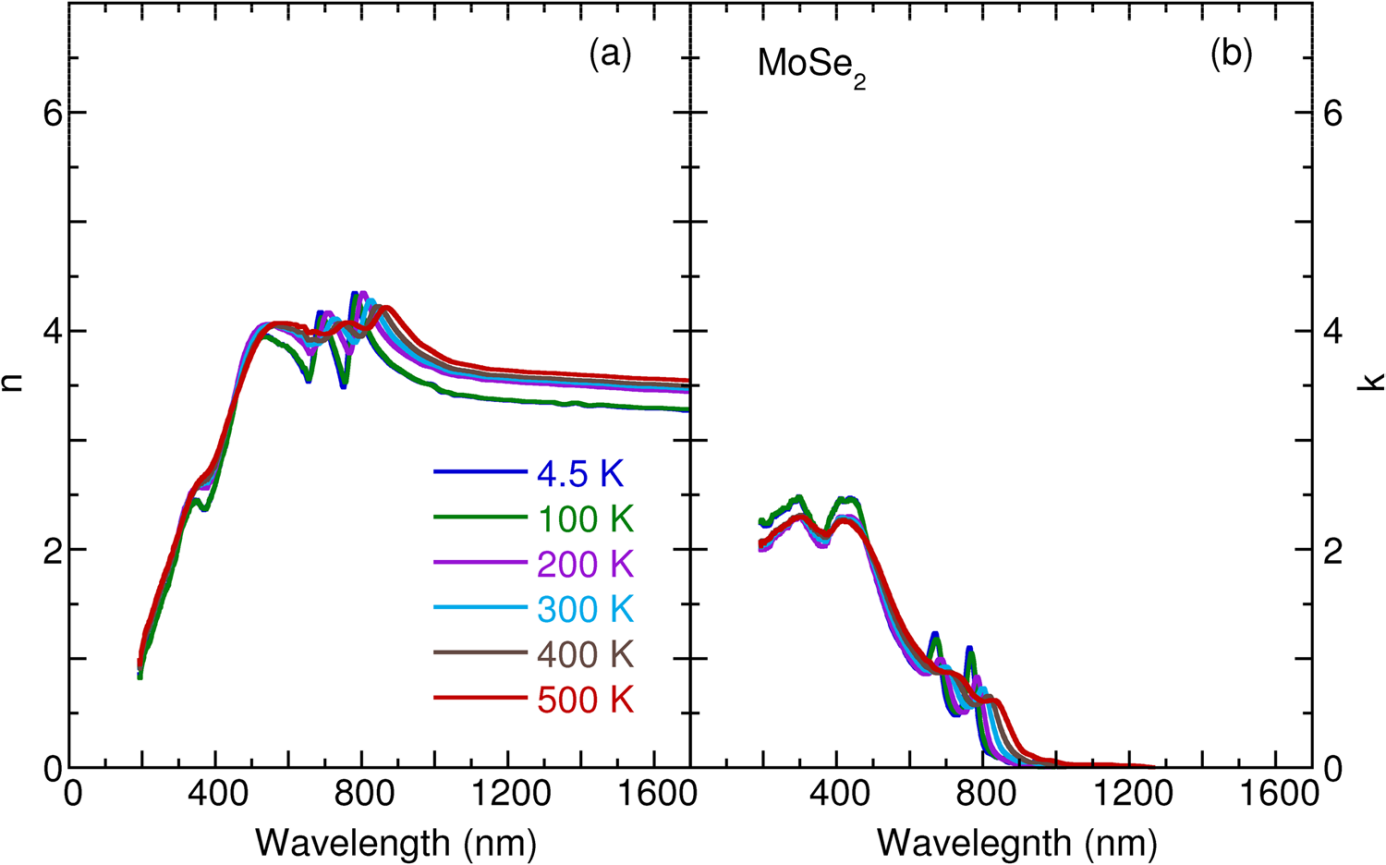
Temperature dependence of (**a**) refractive index *n* and (**b**) extinction coefficient *k* of monolyaer ${\text {MoSe}}_2$.

**Figure 3:**
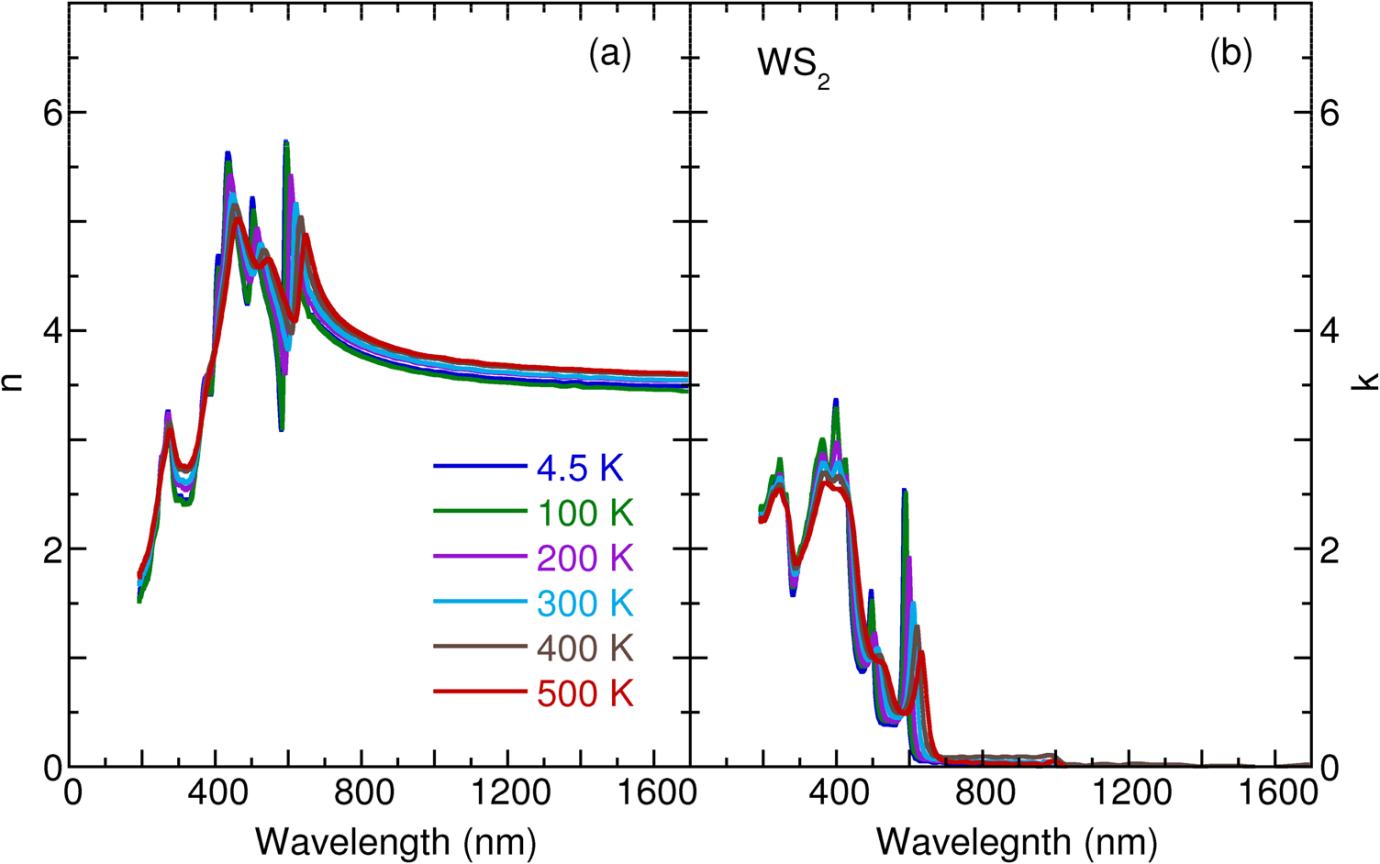
Temperature dependence of (**a**) refractive index *n* and (**b**) extinction coefficient *k* of monolyaer ${\text {WS}}_2$.

**Figure 4:**
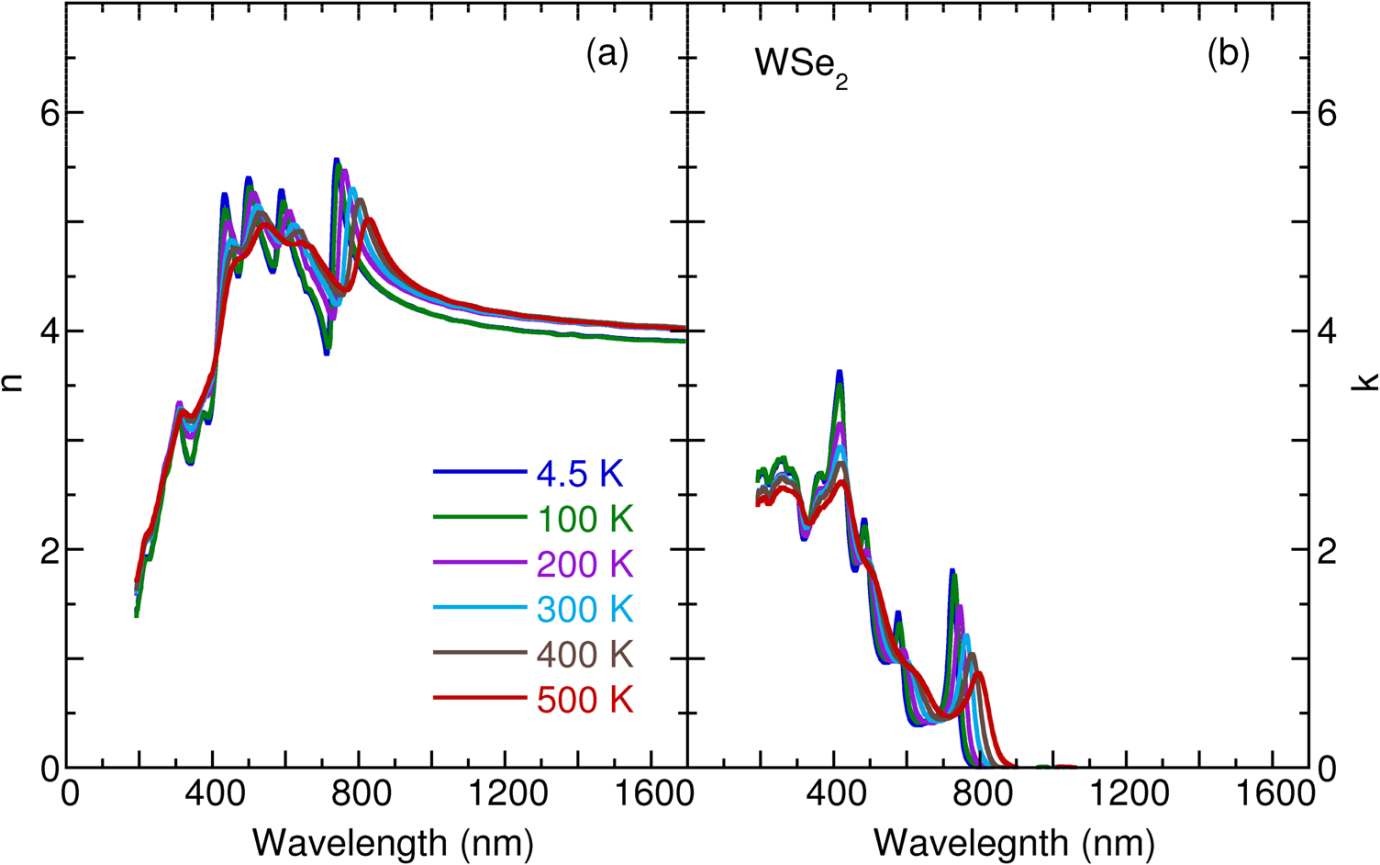
Temperature dependence of (**a**) refractive index *n* and (**b**) extinction coefficient *k* of monolyaer ${\text {WSe}}_2$.

**Figure 5:**
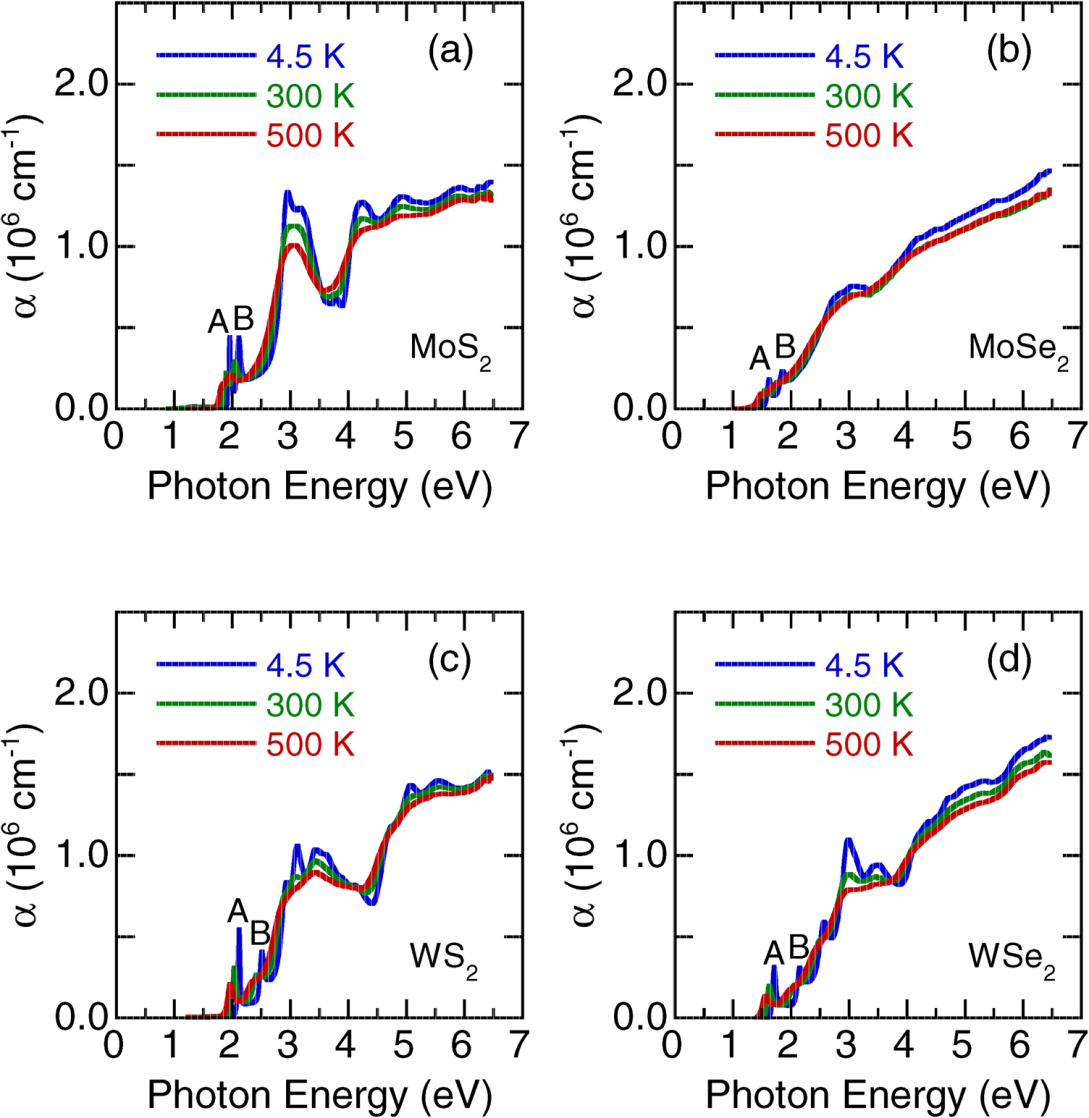
Temperature-dependent optical absorption coefficient $\alpha$ of monolayer (**a**) ${\text {MoS}}_2$, (**b**) ${\text {MoSe}}_2$, (**c**) ${\text {WS}}_2$, and (**d**) ${\text {WSe}}_2$. A and B denote the exciton peaks.

**Figure 6:**
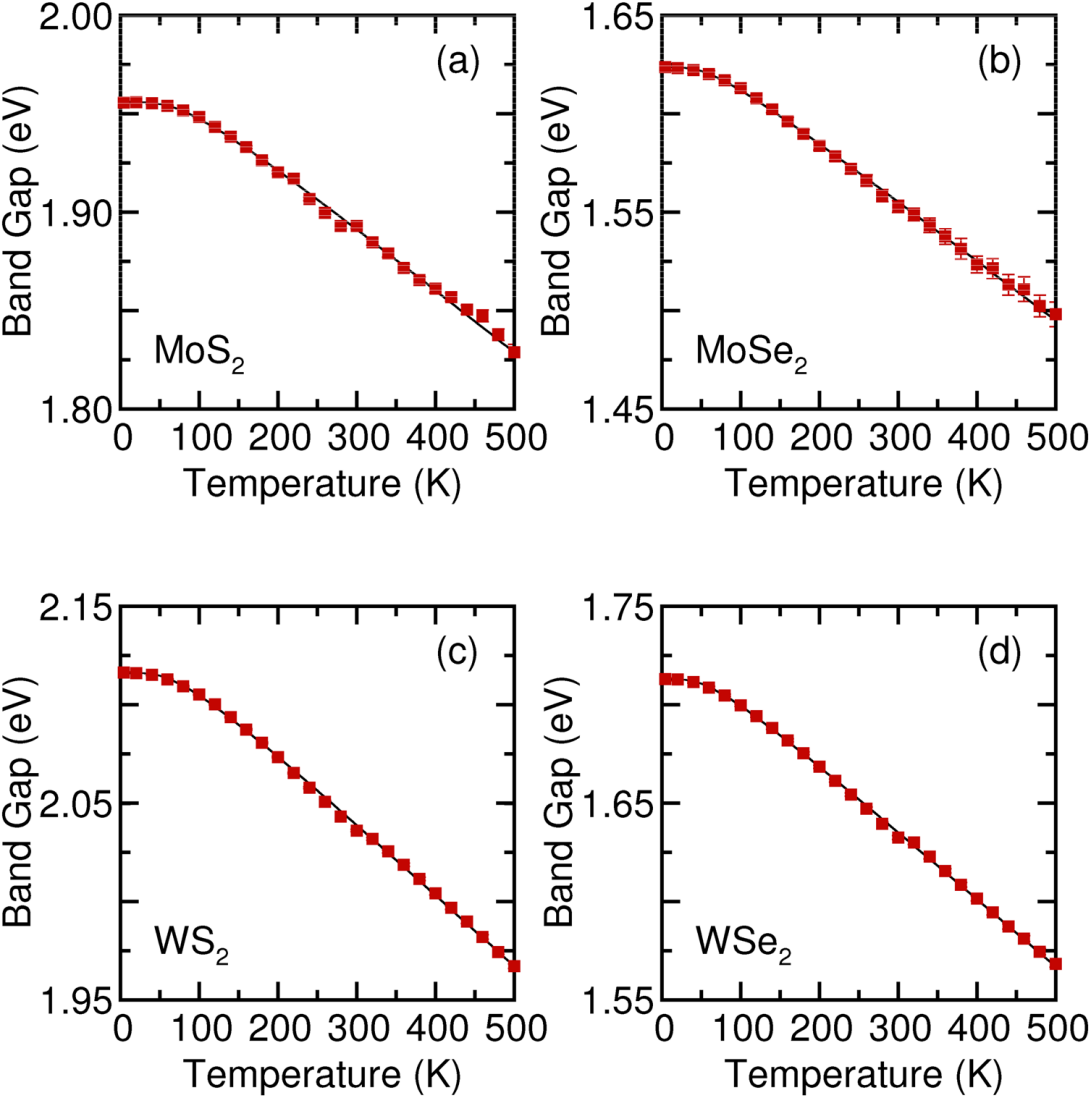
Temperature-dependent optical band gap of monolayer (**a**) ${\text {MoS}}_2$, (**b**) ${\text {MoSe}}_2$, (**c**) ${\text {WS}}_2$, and (**d**) ${\text {WSe}}_2$. The thin solid lines are the results of the fitting using the Bose–Einstein model (Eq. 1).

**Figure 7:**
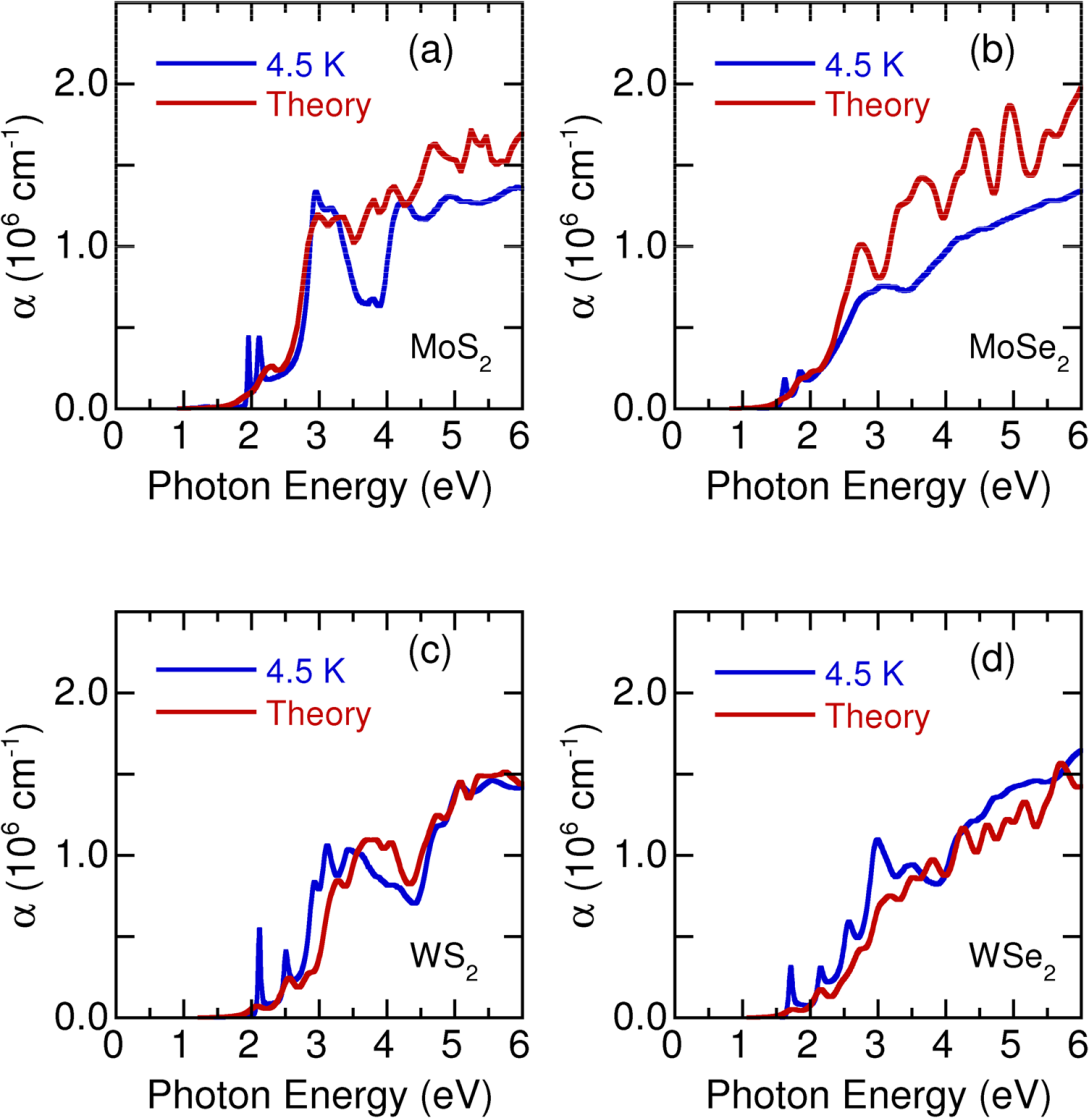
Experimental optical absorption coefficient of monolayer (**a**) ${\text {MoS}}_2$, (**b**) ${\text {MoSe}}_2$, (**c**) ${\text {WS}}_2$, and (**d**) ${\text {WSe}}_2$ at 4.5 K and the theoretical calculation curves.

**Figure 8:**
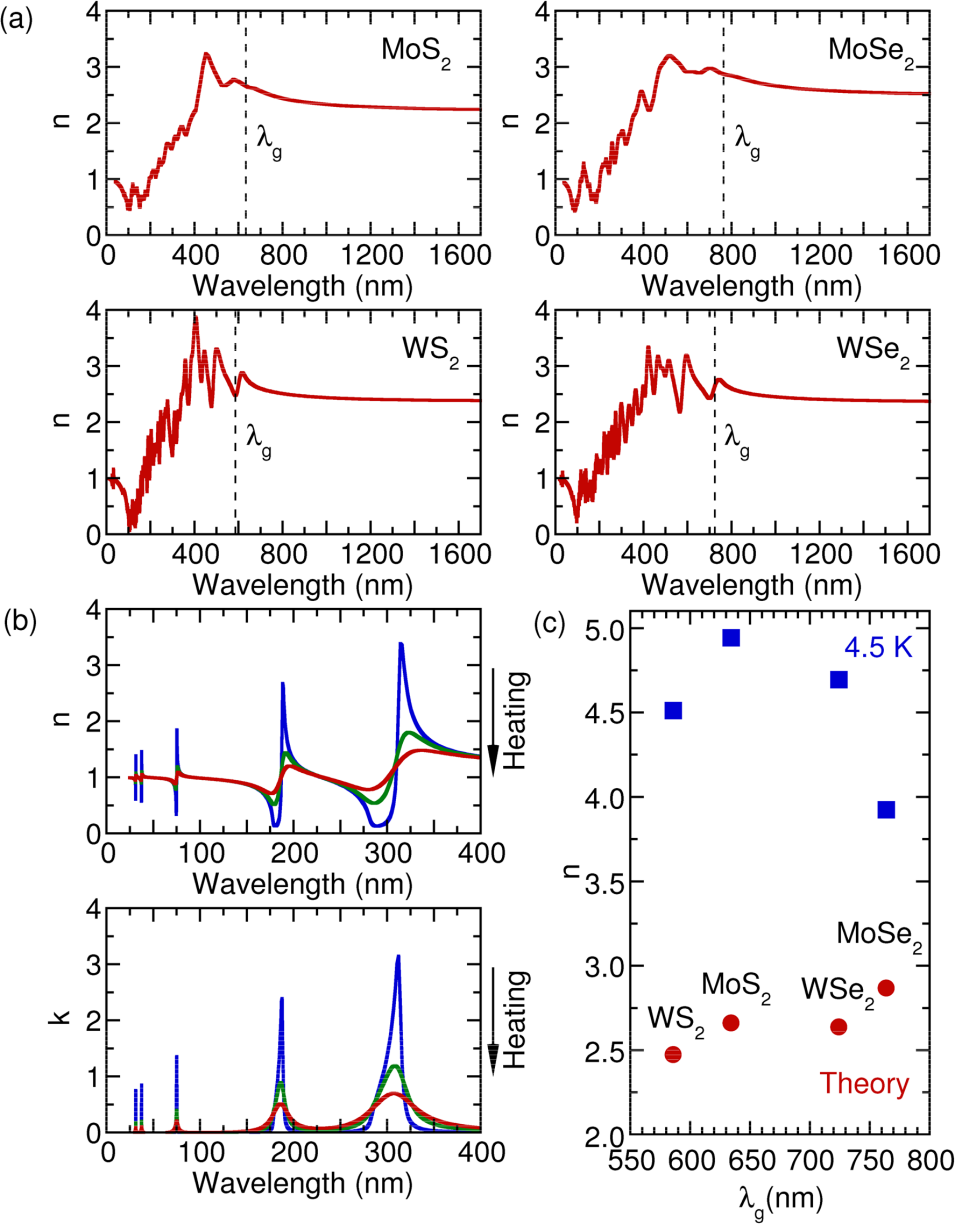
(**a**) The calculated refractive index *n* of monolayer TMDs. (**b**) Refractive index *n* and extinction coefficient *k* as a function of wavelength in dipole oscillator model with multiple resonances. The solid line with arrow in (**b**) indicates the increasing temperature. (**c**) *n* as a function of band gap wavelength $\lambda _g$.

## Results and discussion

Figures 1, 2, 3 and 4 detail the observed temperature dependence of refractive index *n* and extinction coefficient *k* of monolayer ${\text {MoS}}_2$, ${\text {MoSe}}_2$, ${\text {WS}}_2$, and ${\text {WSe}}_2$ as a function of the wavelength from 193 to 1700 nm. The spectra were almost identical by rotating the sample’s azimuthal orientation of $45^\circ$ and $90^\circ$ shown in the Supplementary Figs. 1 and 2, indicating the in-plane isotropic optical properties of monolayer TMDs. These data are necessary to understanding the optical properties of photonic devices for integrated optics and solar cell applications at various temperatures and wavelengths. For these four TMDs, the *n* increases substantially with an increase in wavelength in the spectral range from 193 to 420 nm; it then approaches several maxima, which correspond to the anomalous dispersion regime^33^, and finally decreases with the wavelength until 1700 nm. Moreover, the *n* slightly increases with an increase in temperature in the near-infrared region. The thermo-optic coefficients $\partial n/\partial T$ at room temperature are $6.1 \times 10^{-5} {\hbox {K}}^{-1}$, $1.7 \times 10^{-4} {\hbox {K}}^{-1}$, $1.3 \times 10^{-4} {\hbox {K}}^{-1}$, and $2.6 \times 10^{-4} \, {\hbox {K}}^{-1}$ at a wavelength of 1200 nm for monolayer ${\text {MoS}}_2$, ${\text {MoSe}}_2$, ${\text {WS}}_2$, and ${\text {WSe}}_2$, respectively. The small increase of *n* as a function of temperature is ascribed to the enhanced electron–phonon interaction with an increase in temperature, as observed for other semiconductors, such as Si, GaAs, and InP^34^. It is worth noting that a small jump in *n* between 100 and 200 K in the case of monolayer ${\text {MoSe}}_2$ and ${\text {WSe}}_2$ could be associated with the activation of the phonon modes below $200 \, {\hbox {cm}}^{-1}$^35^. In Figs 1, 2, 3 and 4, the extinction coefficient spectra exhibit several strong absorption peaks below 800 nm. These absorptions show a redshift trend with an increase in temperature, which agrees well with semiconductors, such as GaAs, InP, AlN, and GaN^36,37^. A detailed analysis of temperature-dependent optical absorptions is provided later.

In Fig. 5, we depict the observed optical absorption coefficient of monolayer ${\text {MoS}}_2$, ${\text {MoSe}}_2$, ${\text {WS}}_2$, and ${\text {WSe}}_2$ at 4.5, 300, and 500 K, respectively. The absorption can be divided into (1) a region of photon energy (1.0–2.5 eV), which is understood as excitonic transitions on a relatively low absorption background, and (2) a region of strong absorption at higher photon energies. The absorption features of monolayer ${\text {WSe}}_2$ above 2.5 eV are more complex than others due to strong effects of the Se *p* orbitals^38^. We fitted these absorption spectra using standard Lorentzian functions. Details of the fitting parameters at 4.5 K are provided in Supplementary Tables 1–4. The error bars in the Lorenzian fit for the measured optical absorption data are approximately 0.5%. At 4.5 K, two peaks were assigned as A and B excitons^17^ at approximately 1.96 and 2.12 eV, 1.62 and 1.86 eV, 2.12 and 2.51 eV, and 1.71 and 2.16 eV for the monolayer ${\text {MoS}}_2$, ${\text {MoSe}}_2$, ${\text {WS}}_2$, and ${\text {WSe}}_2$, respectively. The A and B excitons originate from the spin-split direct-gap transitions at the *K* point of the Brillouin zone. Thus, the spin–orbit splitting of the valence-band at the *K* point can be approximately estimated from the energy difference between the A and B exciton peaks. The measured spin–orbit splitting energy for $T = 4.5 \, \hbox {K}$ was 160, 204, 390, and 450 meV for monolayer ${\text {MoS}}_2$, ${\text {MoSe}}_2$, ${\text {WS}}_2$, and ${\text {WSe}}_2$, respectively. These results are in agreement with the other theoretical predictions^27,28,30,31,39–41^. The measured spin–orbit splitting energy at 4.5 K was greatly enhanced in the tungsten dichalcogenides than the molybdenum counterparts. The giant spin–orbit splitting in monolayer ${\text {WS}}_2$ and ${\text {WSe}}_2$ benefits them in potential applications in spintronics.

The peak positions of A and B excitons were shifted to lower energies, and their resonance linewidth increased with an increase in temperature. As illustrated in Fig. 6, we extracted the value of the optical band gap from the A exciton peak position as a function of temperature. The optical band gap describes the energy required to create an exciton, a correlated two-particle electron–hole pair, via optical absorptions for the A and B energy gaps^42–45^. The difference in the optical band gap of monolayer TMDs can be attributed to the difference in the chemical compositions, for example, the difference between electro-negativity (see Supplementary Fig. 3). The optical band gap decreases with an increase in temperature. The temperature dependence of the B exciton peak position is described in Supplementary Fig. 4. The observed redshift value of the optical band gap with an increase in temperature in semiconductors can be described using the Bost–Einstein model^46^:

1 $$\begin{aligned} E_g(T) = E_g(0) - {2a_B \over {[\mathrm{exp}(\Theta _B / T) - 1]}}, \end{aligned}$$

where $E_g(0)$ is the band gap energy at 0 K, $a_B$ represents the strength of the electron–phonon interactions, and $\Theta _B$ is the average phonon temperature. In this model, the electron–phonon interactions are responsible for the shrinkage in the band gap with an increase in temperature^46^. In Fig. 6, the fitted $E_g(T)$ values are indicated by solid lines. As in Fig. 6, the Bose–Einstein model reproduces the temperature dependence of the optical band gap in the monolayer TMDs. The parameters used to fit the temperature-dependent optical band gap are listed in Table 1. The error bars in the fit for temperature-dependent optical band gap are approximately 0.5%. Notably, in monolayer ${\text {MoSe}}_2$, a previous ellipsometric study determined only the linear temperature dependence of the optical band gap for 300–800 K^22^.

**Table 1:** The fitting parameters of the Bose–Einstein model.

	${\text {MoS}}_2$	${\text {MoSe}}_2$	${\text {WS}}_2$	${\text {WSe}}_2$
${\text {E}}_g$(0) (eV)	1.99	1.65	2.15	1.74
${\text {a}}_B$ (meV)	36	26	37	29
$\Theta _B$ (K)	225	170	200	165

Figure 5 presents the absorption spectra for the higher photon energy regions (> 2.5 eV) of monolayer ${\text {MoS}}_2$, ${\text {MoSe}}_2$, ${\text {WS}}_2$, and ${\text {WSe}}_2$ , which have a considerably higher intensity than those for the A and B exciton peaks. The assignments of the higher photon energy spectra remain controversial^5,27^. One study reported that these absorption bands were caused by the split excitons A’ and B’^5^, whereas another reported that these absorption peaks were related to strongly bound excitons^27^. Nevertheless, with an increase in temperature, all absorption peaks shifted to lower photon energies and broadened. To understand the origin and nature of these high energy absorption bands, we calculated the electronic band structure and optical absorption spectra^43^. In Fig. 7, the experimental optical absorption coefficient $\alpha$ (blue lines) at 4.5 K is compared with the calculated $\alpha$ (red lines) for the monolayer TMDs. Experimental *n* and *k* of monolayer ${\text {MoS}}_2$, ${\text {MoSe}}_2$, ${\text {WS}}_2$, and ${\text {WSe}}_2$ at 4.5 K were also compared with the theoretical calculation curves(see Supplementary Figs. 5–8). The peak positions and general trend of $\alpha$ in the experiment and calculation agree reasonably well. Each absorption peak corresponds to one electron–photon resonance, and all the peaks give rise to multiple resonances. The calculated results indicated that the observed strong optical absorption around 3.0 eV in the monolayer TMDs is a result of the band gap ($\sim$ 2.97 eV) at the *M* point (the edge center point of the Brillouin zone). Usually, the *M* point corresponds to the van-Hove singularity point of two-dimensional materials, giving rise to a logarithmically diverging electronic density of states, which results in a large optical absorption value. The optical absorptions above 3.0 eV arose from the nested bands between the $\Gamma$ and *K* points, in which the joint density of states became large. We will present a more detailed analysis of the photon energy dependence of optical absorption and refractive index later within the dipole oscillator model^47^.

In Fig. 8, we present the calculated refractive index as a function of the wavelength of light. The calculated *n* appropriately reproduces the experimental data in Figs. 1, 2, 3 and 4. Two characteristics can be observed. One is that *n* decreases with wavelength when $\lambda \ge \lambda _g$ ($\lambda _g$ corresponds to the wavelength of the optical band gap), which is a consequence of the Kramers–Kronig relations between the real part *n* and imaginary part *k* of the complex refractive index^47^. The other is that *n* increases with wavelength $\lambda$ but with many oscillations when $\lambda \le \lambda _g$. To better understand the oscillations, we employ the dipole oscillator model for multiple resonances to have a qualitative view^33^. Within the model, the relative dielectric index $\varepsilon _r$ has the following form:

2 $$\begin{aligned} \varepsilon _r(\omega ) = 1 + \frac{Ne^2}{m_0 \varepsilon _0} \sum _j \frac{1}{(\omega _j^2 - \omega ^2 - i \gamma _j \omega )}, \end{aligned}$$

in which *N* is the number of dipoles per unit volume, $m_0$ is the reduced mass of the dipole, $\omega _j$ is the resonant frequency due to the presence of one dipole, and $\gamma _j$ is the damping rate. Starting at the lowest wavelength and gradually working up to the band gap wavelength, we assume the existence of three typical resonance wavelengths as depicted in Fig. 8b. At the lowest wavelengths, or the highest frequencies, the electrons cannot follow the alternating electric fields, which provides dielectric constant unity. When we increase the wavelength and through these resonances, we observe the characteristic wavelength for $\omega = \omega _j$ dependence of the Lorentz oscillator, with a peak in the absorption spectrum and an oscillating refractive index. Between the two resonances peaks, absorption goes to zero, and *n* becomes a constant. The constant value of *n* grows with an increase in resonances when the wavelength increases. Anomalous dispersion can be appropriately interpreted within the dipole oscillator model due to multiple resonance frequencies. Also worth noting is the linear relationship between the refractive index $n_g$ and $\lambda _g$ at the band gap for the TMDs materials, as seen in Fig. 8c. Such behavior can also be understood as indicating the Kramers–Kronig relations between *n* and *k*, usually taking the form $n = 1 + \frac{2\gamma k \lambda _g}{\pi ^2 c}$.

The difference in the *n* between the experimental results and calculations in Fig. 8c can be explained as follows: (1) the results from the dipole oscillator model, as seen in Fig. 8b, indicates that the *n* is cumulative, and the plateau value increases stepwise from the highest frequencies to the band gap energy level. To accurately calculate the frequency-dependent dielectric function and optical absorption, an appreciable number of empty conduction states is required; however, in real calculations this number is limited, causing a decrease in the number of plateau and an underestimation of the *n* . (2) Local field effects (changes in the cell periodic part of potential) were neglected in the present calculation. The origins for the anomalous result of monolayer ${\text {MoSe}}_2$ are unclear at this time. A more accurate evaluation can be applied using either density functional perturbation theory^48^ or the GW method^49^, which can be our next consideration.

To obtain an insight into the temperature dependence of the *n* in Figs. 1, 2, 3 and 4, we studied the temperature effect on the $\gamma _j$ parameter in the dipole oscillator model, as presented in Fig. 8b. With an increase in temperature, the resonance absorption peaks decrease and broaden, as does the oscillating element of the *n*. However, the plateau of *n* does not change with temperature. The agreement between the model and experimental data indicates increasing numbers of temperature-induced phonons, which gives rise to enhanced damping $\gamma _j$.

## Summary

We investigated the temperature-dependent optical constants of monolayer ${\text {MoS}}_2$, ${\text {MoSe}}_2$, ${\text {WS}}_2$, and ${\text {WSe}}_2$ using spectroscopic ellipsometry. The absorption emerging in the extinction coefficient spectrum indicated that the monolayer TMDs have a direct band gap with a large exciton binding energy. With a decrease in temperature, the refractive indices decreased in the near-infrared region. The thermo-optic coefficients at room temperature ranged from $6.1 \times 10^{-5}$ to $2.6 \times 10^{-4} \, {\hbox {K}}^{-1}$ at a wavelength of 1200 nm. The optical band gap increased due to the suppression of electron–phonon interactions. The data presented in this study provide standard information for the design and fabrication of monolayer TMDs-based photonic devices for integrated optics and solar cell applications at various temperatures.

## Method

### Experiment

Monolayer ${\text {MoS}}_2$, ${\text {MoSe}}_2$, ${\text {WS}}_2$, and ${\text {WSe}}_2$ were grown on *c*-axis cut sapphire substrates by ambient-pressure chemical vapor deposition with the seeding of perylene-3,4,9,10-tetracarboxylic acid tetrapotassium salt^50,51^. The details of sample preparation were as reported previously in Figure S2 and Table T1 of Ref.^50^. That these thin films were single-layer was verified by atomic force microscopy^50^ and Raman scattering spectroscopy (see Supplementary Fig. 9)^52,53^. Spectroscopic ellipsometric spectra were measured for multiple angles of incidence between $60^\circ$ and $75^\circ$ by using a J. A. Woollam Co. M-2000U ellipsometer over the spectral range from 0.73 to 6.42 eV. For the ellipsometric measurements, the multiple angles of incidence were chosen so as to maximize the difference between the intensities of the *p*-wave and the *s*-wave. In order to meet this condition, one should use an angle which is near the Brewster angle for the substrate. This is particularly important for monolayer TMDs. Because the Brewster angle for the sapphire substrate in the visible region is about $60.5^{\,\circ }$ , the ellipsometric spectra of monolayer TMDs were measured for multiple angles of incidence between $60^\circ$ and $75^\circ$. The complex optical constants were obtained through spectroscopic ellipsometry using the stacked layer model (sapphire substrate/thin film/surface roughness/air ambient structure). The parameters of the model used to fit the raw ellipsometry data are listed in Table 2. The values of the mean square error (MSE) are 0.968, 1.151, 0.718, and 0.476 for monolayer ${\text {MoS}}_2$, ${\text {MoSe}}_2$, ${\text {WS}}_2$, and ${\text {WSe}}_2$, respectively. The *c*-axis cut sapphire substrate eliminates the inherent birefringent properties of the crystal. We adopted the optical constants of the *c*-axis cut sapphire substrate in the stacked layer model, which takes into account the anisotropic properties of sapphire substrate. The multiple angle of incidence spectroscopic ellipsometry is not sensitive to monolayer TMDs’ *c*-axis optical response because of their one-atom-thick layer. Thus, we focus on the in-plane optical properties of monolayer TMDs. Moreover, the surface roughness model automatically mixed (50/50) monolayer TMDs with vacuum (n = 1), which is similar to the approach of Bruggeman effective-medium^54^. The only fitting parameter is the thickness of surface roughness. The optically detected surface roughness could arise from the gradual oxidation along the grain boundaries and the adsorption of organic contaminants^55^. The layer thickness of TMDs obtained from the spectroscopic ellipsometry analysis was 0.6–0.7 nm, which is in good agreement with the literature data^2,3,5^. The independently measured experimental data at different incidence angles and the modeled curves are in good agreement (see Supplementary Figs. 10–14). Spectroscopic ellipsometric spectra were also measured between 4.5 and 500 K, for which the ellipsometer was equipped with an ultrahigh-vacuum continuous-flow liquid helium cryostat.

**Table 2:** Parameters of the stacked layer model fit for the monolayer TMDs. All units are in nm.

	${\text {MoS}}_2$	${\text {MoSe}}_2$	${\text {WS}}_2$	${\text {WSe}}_2$
Thickness	0.71 ± 0.07	0.70 ± 0.07	0.65 ± 0.07	0.62 ± 0.06
Surface roughness	0.07 ± 0.01	0.16 ± 0.02	0.07 ± 0.01	0.07 ± 0.01

### Theoretical model

The electronic band structure and phonon dispersion relationships of monolayer ${\text {MoS}}_2$, ${\text {MoSe}}_2$, ${\text {WS}}_2$, and ${\text {WSe}}_2$ were calculated using first-principles density functional theory within local density approximation as implemented in the Quantum-Espresso code^56^. We used the Perdew–Zunger form of the exchange-correlation functional in the local density approximation. We used the primitive cell for the calculations. The interaction between electrons and ions was described by projector augmented-wave (PAW) pseudopotentials^57,58^ with a plane-wave cutoff energy of 65 Ry. Fully relativistic pseudopotentials derived from an atomic Dirac-like equation^59^ were employed to calculate the electronic band structure with spin–orbit interaction considered. The atomic structure was optimized with an atomic force of less than $10^{-5}$ Ry/Bohr. A *k*-mesh of $12 \times 12 \times 1$ under the Monkhorst–Pack scheme^60^ was used to sample the Brillouin zone. The phonon dispersion relationship of monolayer TMDs was calculated using density functional perturbation theory^61^.

The electronic band structures of monolayer TMDs were given in our previous results and can be referred to Ref.^35,52,53^. The frequency-dependent dielectric matrix was calculated using the electronic ground state. The imaginary part was determined by a summation over empty states using the equation^62^:

3 $$\begin{aligned} \varepsilon ''_{\alpha \beta }(\omega ) = \frac{4\pi ^2 e^2}{\Omega } lim_{q \rightarrow 0} \sum _{c,v,k} 2w_k \delta (\epsilon _{ck} - \epsilon _{vk} -\hbar \omega ) \langle u_{c k+e_{\alpha }q} | u_{vk}\rangle \langle u_{c k+e_{\beta }q} | u_{vk} \rangle ^*, \end{aligned}$$

where $\Omega$ is the volume of the primitive cell, $w_k$ is the *k*-point weight defined such that they sum to 1 for occupied states, *c* and *v* refer to conduction and valence band states, respectively, and ${\text {u}}_{nk}$ ($n = c$, *v*) is the periodic part of the orbitals at each *k* point. The vectors ${\text {e}}_{\alpha }$ are unit vectors for the three Cartesian directions. The real dielectric tensor $\varepsilon '(\omega )$ was obtained using the conventional Kramers–Kronig transformation. Considering that the calculation of the superlattice dielectric function in Eq. (3) can be highly sensitive to interlayer spacing along the perpendicular axis^63^, we have calculated the electric fields required to compensate the dipole of the system at each iteration of the self-consistency cycle. The potential added to the grid corresponds to that of a dipole layer at the middle of the vacuum layer. For slabs, this exactly compensates the electric fields at the vacuum created by the dipole moment of the system, thus allowing to treat asymmetric slabs and to compute optical properties more properly.

The refractive index and optical absorption spectrum were calculated using the real ($\varepsilon '$) and imaginary ($\varepsilon ''$) parts of the dielectric function as a function of photon energy, following the PAW methodology^62^. The refractive index $n = \sqrt{(\sqrt{\varepsilon '^2 + \varepsilon ''^2} + \varepsilon ')/2}$. The extinction coefficient^64^ $k = \sqrt{(\sqrt{\varepsilon '^2 + \varepsilon ''^2} - \varepsilon ')/2}$. The absorption coefficient $\alpha$ is described by $\alpha = 4 \pi k E /(\textit{hc})$, where *E* is the photon energy, *h* is the Planck constant, and *c* is the speed of light.

## Supplementary information

Supplementary Information.

## Data Availability

The data that support the findings of this study are available from the corresponding author upon reasonable request.
