# Supplementary material for: Temperature-dependent optical constants of monolayer MoS2, MoSe2, WS2, and WSe2: spectroscopic ellipsometry and first-principles calculations
Source: Sci Rep. 2020 Sep 17;10:15282. doi: 10.1038/s41598-020-71808-y (PMC7498615; doi:10.1038/s41598-020-71808-y)
Supplement: Supplementary file 1 — Supplementary Information. [file 41598_2020_71808_MOESM1_ESM.pdf]

Supplementary information

Temperature-dependent optical constants of monolayer MoS_2, $MoSe_2$, WS_2, and WSe_2: Spectroscopic ellipsometry and first-principles calculations

Hsiang-Lin Liu[1,*], Teng Yang[2,3,*], Jyun-Han Chen[1], Hsiao-Wen Chen[1], Huaihong Guo[3,4], Riichiro Saito[3], Ming-Yang Li[5,6], and Lain-Jong Li[6]

[1]*Department of Physics, National Taiwan Normal University, Taipei 11677, Taiwan*

[2]*Shenyang National Laboratory for Materials Science, Institute of Metal Research, Chinese Academy of Sciences, 72 Wenhua Road, Shenyang 110016, China*

[3]*Department of Physics, Tohoku University, Sendai 980-8578, Japan*

[4]*College of Sciences, Liaoning Shihua University, Fushun, 113001, China*

[5]*Research Center for Applied Science, Academia Sinica, Taipei 10617, Taiwan*

[6]*Physical Science and Engineering Division, King Abdullah University of Science and Technology, Thuwal 23955-6900, Kingdom of Saudi Arabia*

*Corresponding author: hliu@ntnu.edu.tw, yangteng@imr.ac.cn

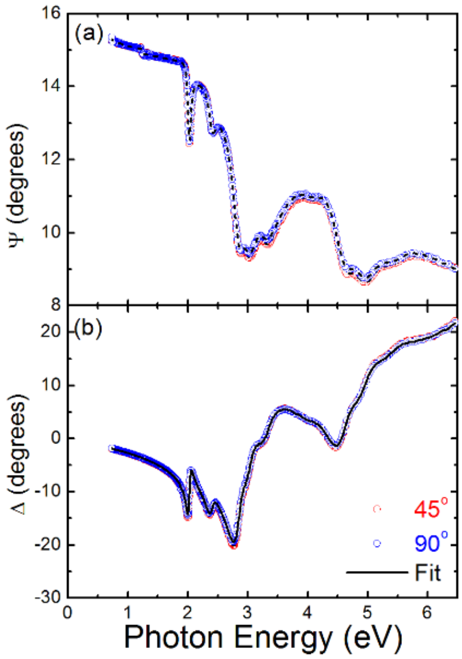

Supplementary Fig. 1 Room temperature experimental at 70° incidence angle and fitting model of ellipsometric parameters of (a) psi (Ψ) and (b) delta (Δ) of monolayer WS_2.

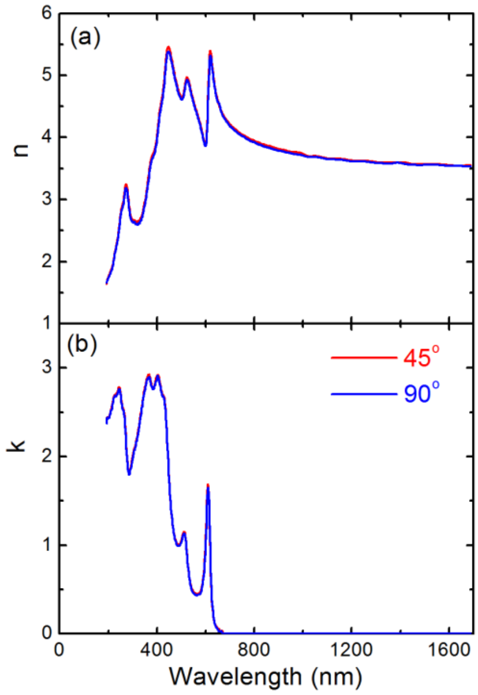

Supplementary Fig. 2 Room temperature (a) refractive index *n* and (b) extinction coefficient *k* of monolayer WS_2 by rotating the sample's azimuthal orientation by 45 degree and 90 degree.

$$\alpha(\omega) = \mathrm{Im}\left[\sum_{j=1}^{N} \frac{\omega_{pj}^2}{\left(\omega_j^2 - \omega^2\right) - i\omega\gamma_j}\right]$$

where ω_j, γ_j, and ω_{pj} are the frequency, damping, and oscillator strength of the jth Lorentzian contributions.

Supplementary Table 1 Parameters of a Lorenzian fit for the measured optical absorption data of monolayer MoS_2 at 4.5 K.

ω_1 (eV)	1.96
γ_1 (eV)	0.02
ω_{p1} (cm^{-1})	770
ω_2 (eV)	2.12
γ_2 (eV)	0.08
ω_{p2} (cm^{-1})	1300
ω_3 (eV)	2.94
γ_3(eV)	0.19
ω_{p3} (cm^{-1})	2800
ω_4 (eV)	3.20
γ_4 (eV)	0.63
ω_{p4} (cm^{-1})	6400
ω_5 (eV)	3.76
γ_5 (eV)	0.15
ω_{p5} (cm^{-1})	936
ω_6 (eV)	4.11
γ_6 (eV)	0.24
ω_{p6} (cm^{-1})	2400
ω_7(eV)	4.30
γ_7 (eV)	0.49
ω_{p7} (cm^{-1})	4100
ω_8 (eV)	4.83
γ_8 (eV)	0.88
ω_{p8} (cm^{-1})	6300
ω_9 (eV)	5.33
γ_9 (eV)	0.92
ω_{p9} (cm^{-1})	5100

ω_{10} (eV)	5.89
γ_{10} (eV)	0.98
ω_{p10} (cm^{-1})	6600

Supplementary Table 2 Parameters of a Lorenzian fit for the measured optical absorption data of monolayer $MoSe_2$ at 4.5 K.

ω_1 (eV)	1.62
γ_1 (eV)	0.03
ω_{p1} (cm^{-1})	500
ω_2 (eV)	1.86
γ_2 (eV)	0.10
ω_{p2} (cm^{-1})	870
ω_3 (eV)	2.65
γ_3(eV)	0.76
ω_{p3} (cm^{-1})	4000
ω_4 (eV)	3.10
γ_4 (eV)	0.83
ω_{p4} (cm^{-1})	4200
ω_5 (eV)	4.14
γ_5 (eV)	1.47
ω_{p5} (cm^{-1})	7500
ω_6 (eV)	5.25
γ_6 (eV)	1.84
ω_{p6} (cm^{-1})	8800

Supplementary Table 3 Parameters of a Lorenzian fit for the measured optical absorption data of monolayer WS_2 at 4.5 K.

ω_1 (eV)	2.12
γ_1 (eV)	0.04
ω_{p1} (cm^{-1})	1130
ω_2 (eV)	2.51
γ_2 (eV)	0.10
ω_{p2} (cm^{-1})	1350
ω_3 (eV)	2.91
γ_3(eV)	0.20
ω_{p3} (cm^{-1})	2520
ω_4 (eV)	3.12
γ_4 (eV)	0.24
ω_{p4} (cm^{-1})	3200
ω_5 (eV)	3.41
γ_5 (eV)	0.37
ω_{p5} (cm^{-1})	3740
ω_6 (eV)	3.64
γ_6 (eV)	0.36
ω_{p6} (cm^{-1})	3125
ω_7(eV)	3.88
γ_7 (eV)	0.40
ω_{p7} (cm^{-1})	2820
ω_8 (eV)	4.17
γ_8 (eV)	0.45
ω_{p8} (cm^{-1})	3250
ω_9 (eV)	4.69
γ_9 (eV)	0.37
ω_{p9} (cm^{-1})	3360
ω_{10} (eV)	5.05
γ_{10} (eV)	0.56
ω_{p10} (cm^{-1})	4920
ω_{11} (eV)	5.59
γ_{11} (eV)	1.04
ω_{p11} (cm^{-1})	7825

Supplementary Table 4 Parameters of a Lorenzian fit for the measured optical absorption data of monolayer WSe_2 at 4.5 K.

ω_1 (eV)	1.71
γ_1 (eV)	0.05
ω_{p1} (cm^{-1})	966
ω_2 (eV)	2.16
γ_2 (eV)	0.13
ω_{p2} (cm^{-1})	2450
ω_3 (eV)	2.56
γ_3(eV)	0.26
ω_{p3} (cm^{-1})	102
ω_4 (eV)	2.96
γ_4 (eV)	0.28
ω_{p4} (cm^{-1})	3300
ω_5 (eV)	3.12
γ_5 (eV)	0.36
ω_{p5} (cm^{-1})	2600
ω_6 (eV)	3.51
γ_6 (eV)	0.69
ω_{p6} (cm^{-1})	5230
ω_7(eV)	4.19
γ_7 (eV)	0.63
ω_{p7} (cm^{-1})	4675
ω_8 (eV)	4.43
γ_8 (eV)	0.44
ω_{p8} (cm^{-1})	2650
ω_9 (eV)	4.70
γ_9 (eV)	0.47
ω_{p9} (cm^{-1})	3600
ω_{10} (eV)	5.00
γ_{10} (eV)	0.59
ω_{p10} (cm^{-1})	4260
ω_{11} (eV)	5.36
γ_{11} (eV)	0.78
ω_{p11} (cm^{-1})	5440

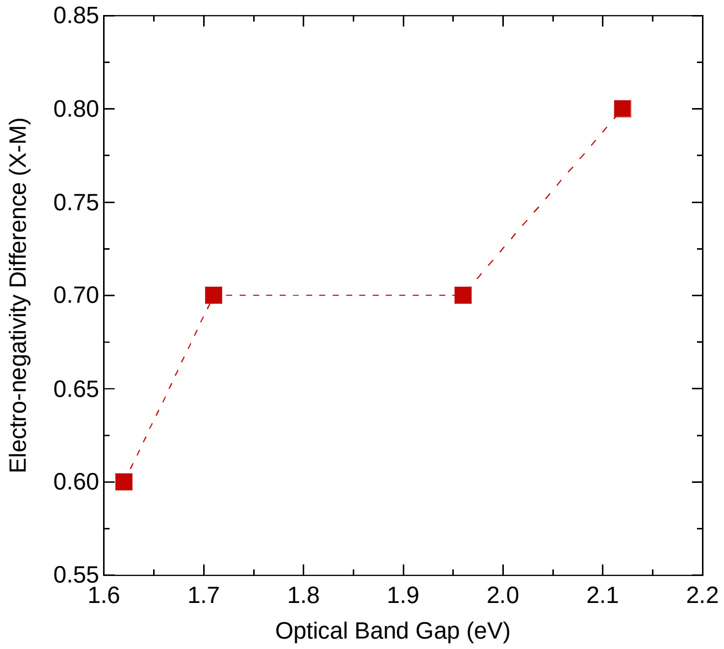

Supplementary Fig. 3 Electro-negativity difference vs. optical band gap of monolayer MoS_2, $MoSe_2$, WS_2, and WSe_2.

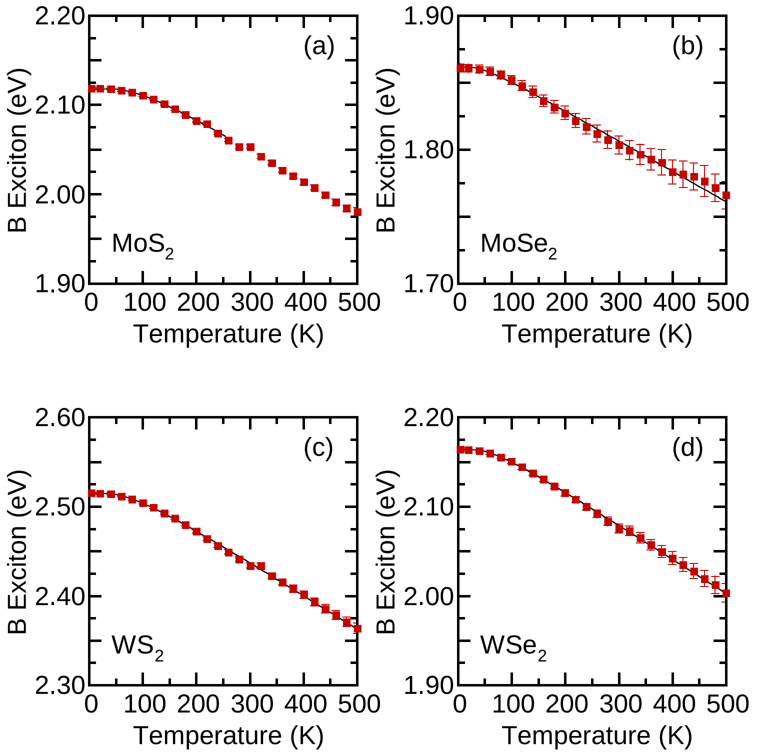

Supplementary Fig. 4 Temperature dependence of peak position of B exciton of monolayer (a) MoS_2, (b) $MoSe_2$, (c) WS_2, and (d) WSe_2. The thin solid lines are the results of the fitting using the Bose-Einstein model.

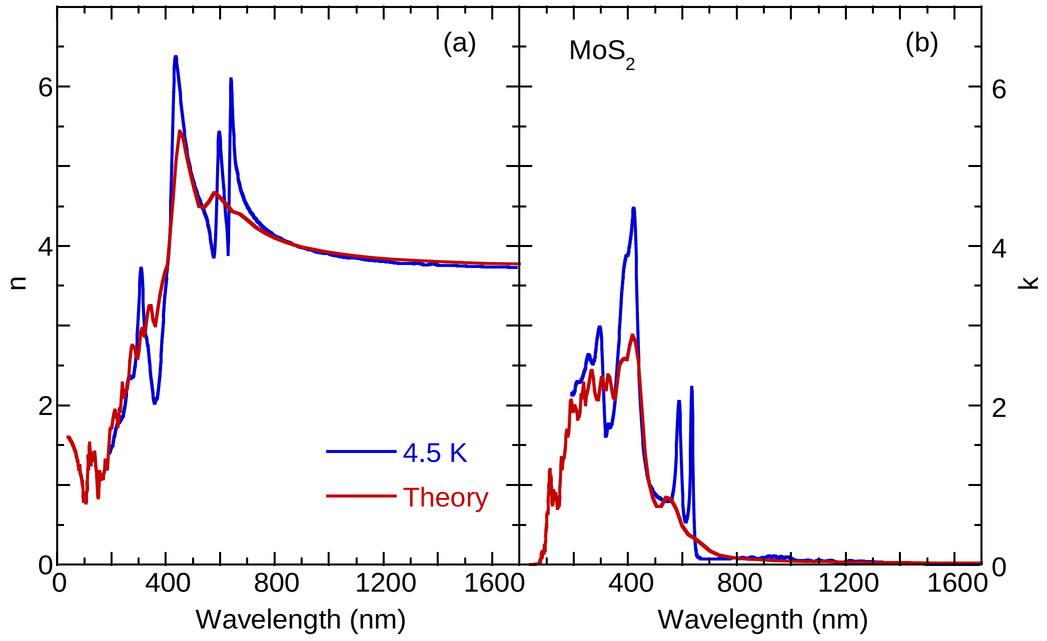

Supplementary Fig. 5 Experimental refractive index *n* and extinction coefficient *k* of monolayer MoS_2 at 4.5 K and theoretical calculation curves. For better comparison, the theoretical *n* spectrum is multiplied by a factor of 1.68.

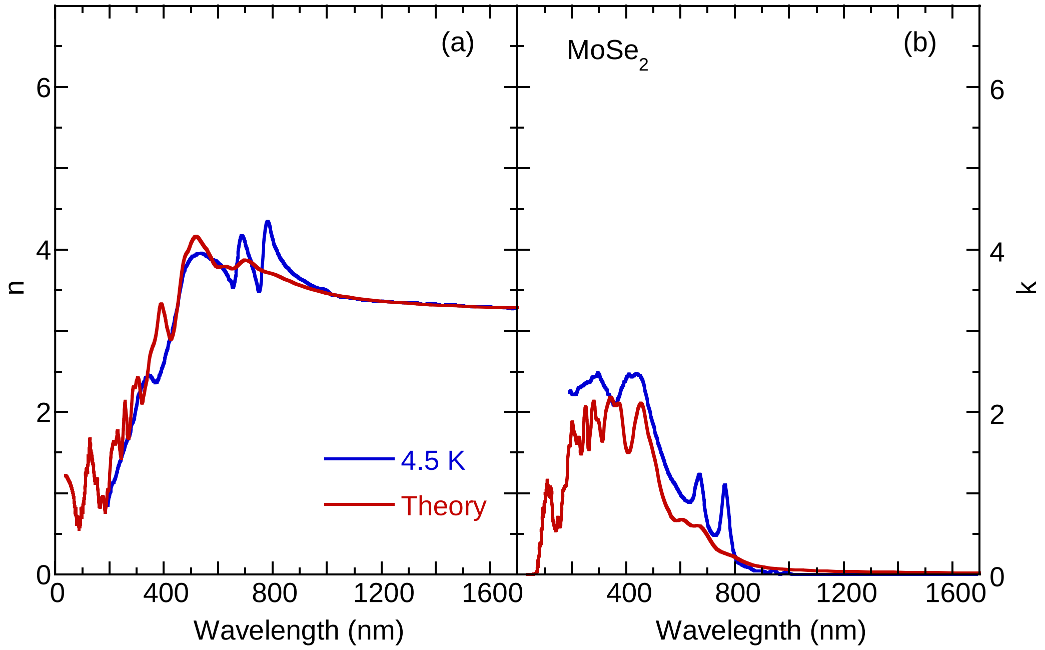

.

Supplementary Fig. 6 Experimental refractive index *n* and extinction coefficient *k* of monolayer $MoSe_2$ at 4.5 K and theoretical calculation curves. For better comparison, the theoretical *n* spectrum is multiplied by a factor of 1.30.

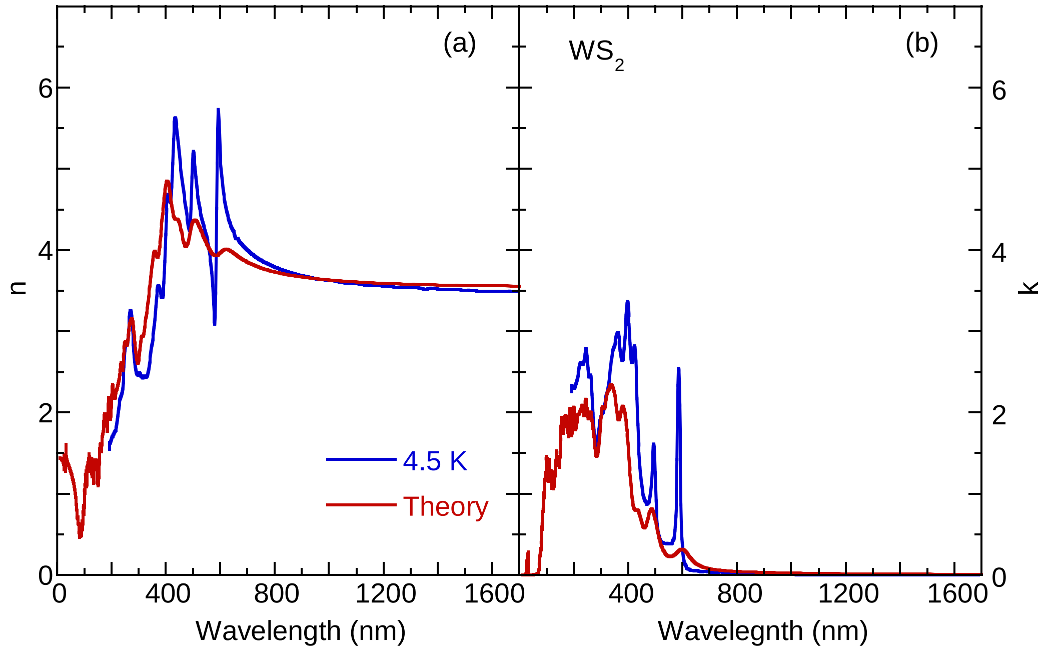

Supplementary Fig. 7 Experimental refractive index *n* and extinction coefficient *k* of monolayer WS_2 at 4.5 K and theoretical calculation curves. For better comparison, the theoretical *n* spectrum is multiplied by a factor of 1.45.

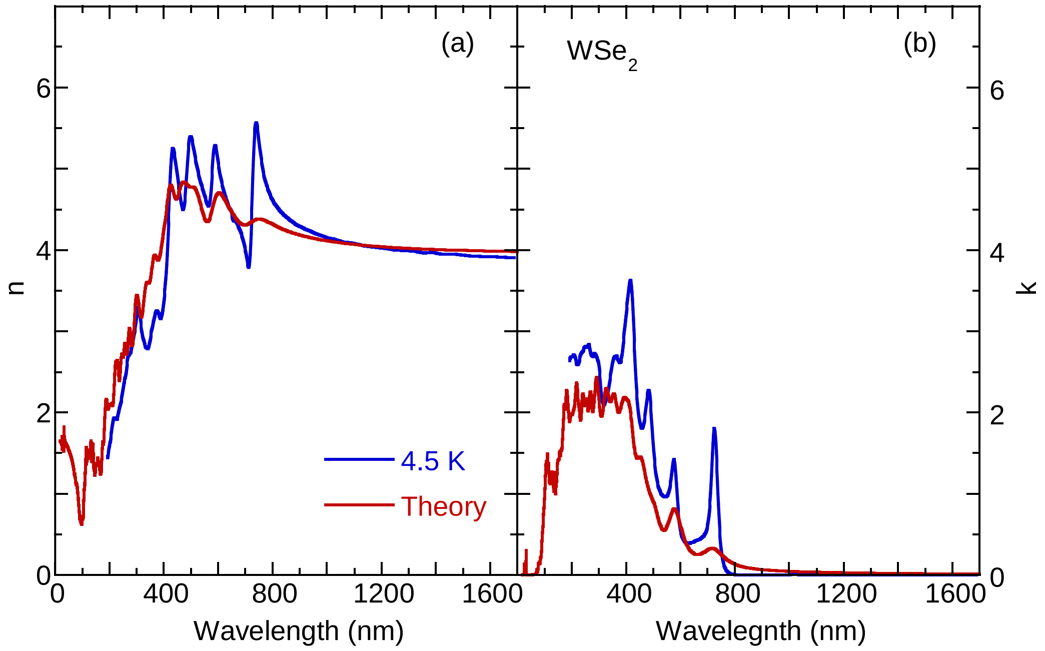

Supplementary Fig. 8 Experimental refractive index *n* and extinction coefficient *k* of monolayer WSe_2 at 4.5 K and theoretical calculation curves. For better comparison, the theoretical *n* spectrum is multiplied by a factor of 1.68.

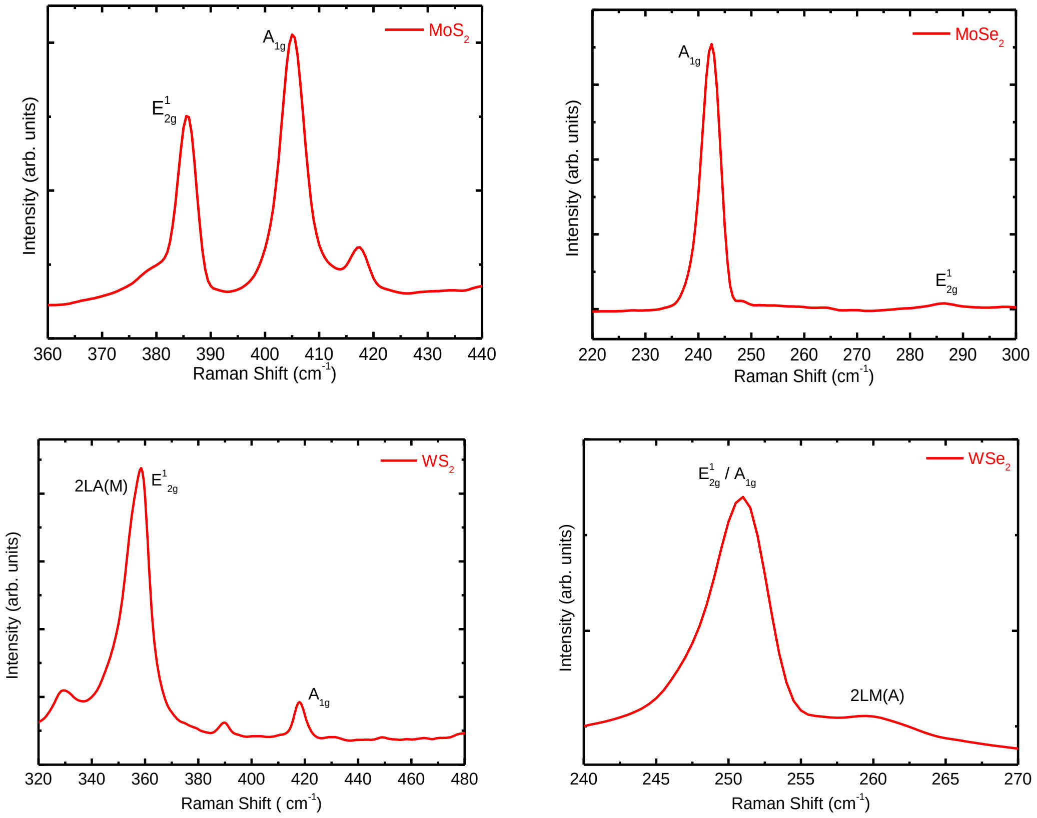

Supplementary Fig. 9 Room temperature Raman scattering spectra of monolayer MoS_2, $MoSe_2$, WS_2, and WSe_2 excited by a 532 nm laser line. These spectra indicated a single-layer signature [52,53].

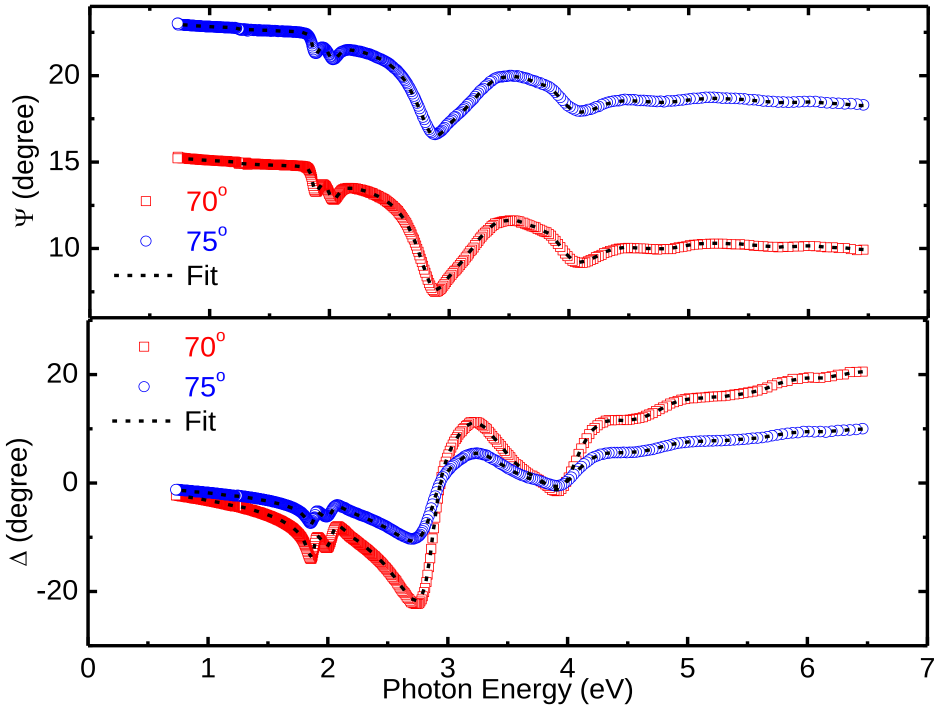

Supplementary Fig. 10 Room temperature experimental at 70° and 75° incidence angles and fitting model of ellipsometric parameters of psi (Ψ) and delta (Δ) of monolayer MoS_2.

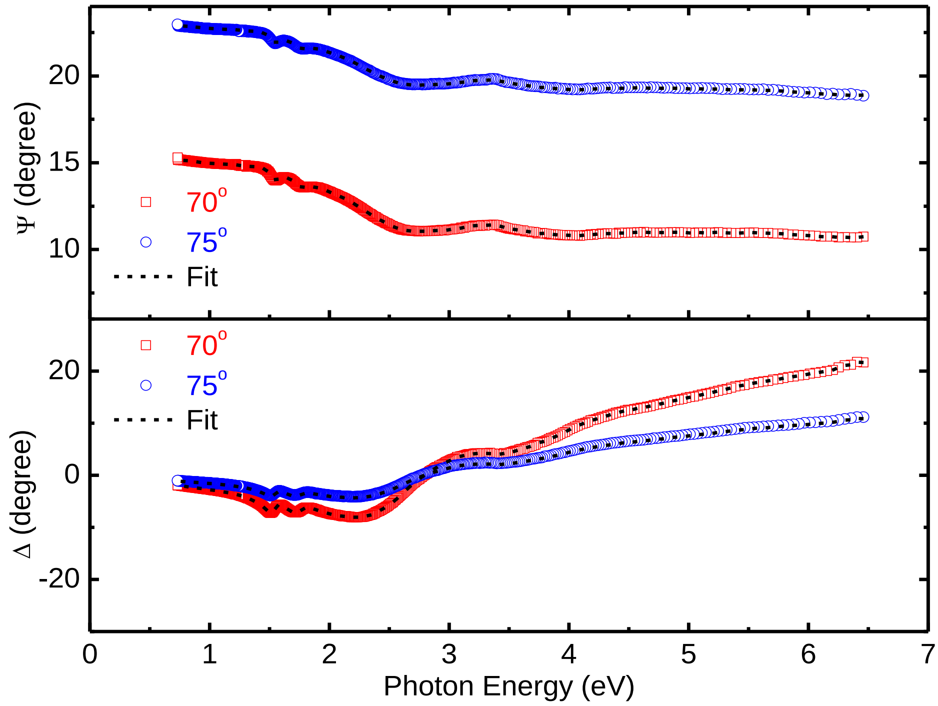

Supplementary Fig. 11 Room temperature experimental at 70° and 75° incidence angles and fitting model of ellipsometric parameters of psi (Ψ) and delta (Δ) of monolayer $MoSe_2$.

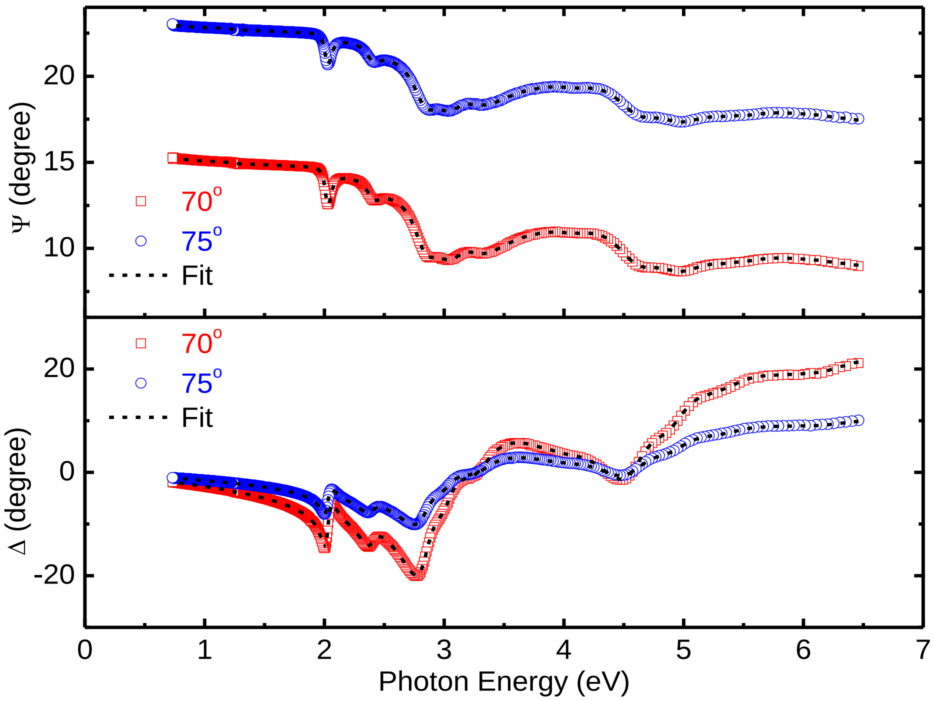

Supplementary Fig. 12 Room temperature experimental at 70° and 75° incidence angles and fitting model of ellipsometric parameters of psi (Ψ) and delta (Δ) of monolayer WS_2.

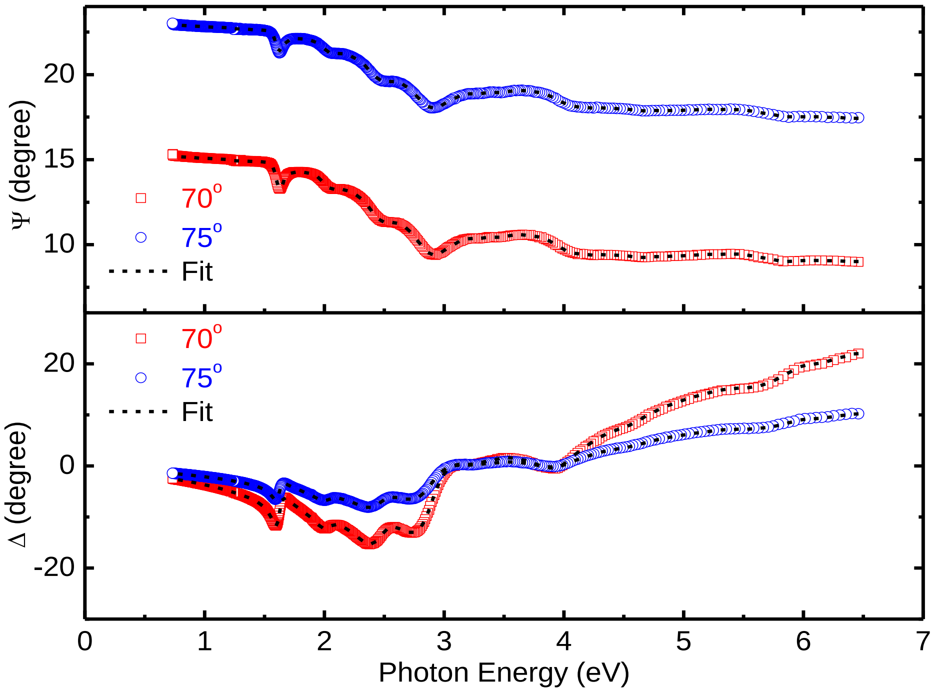

Supplementary Fig. 13 Room temperature experimental at 70° and 75° incidence angles and fitting model of ellipsometric parameters of psi (Ψ) and delta (Δ) of monolayer WSe_2.

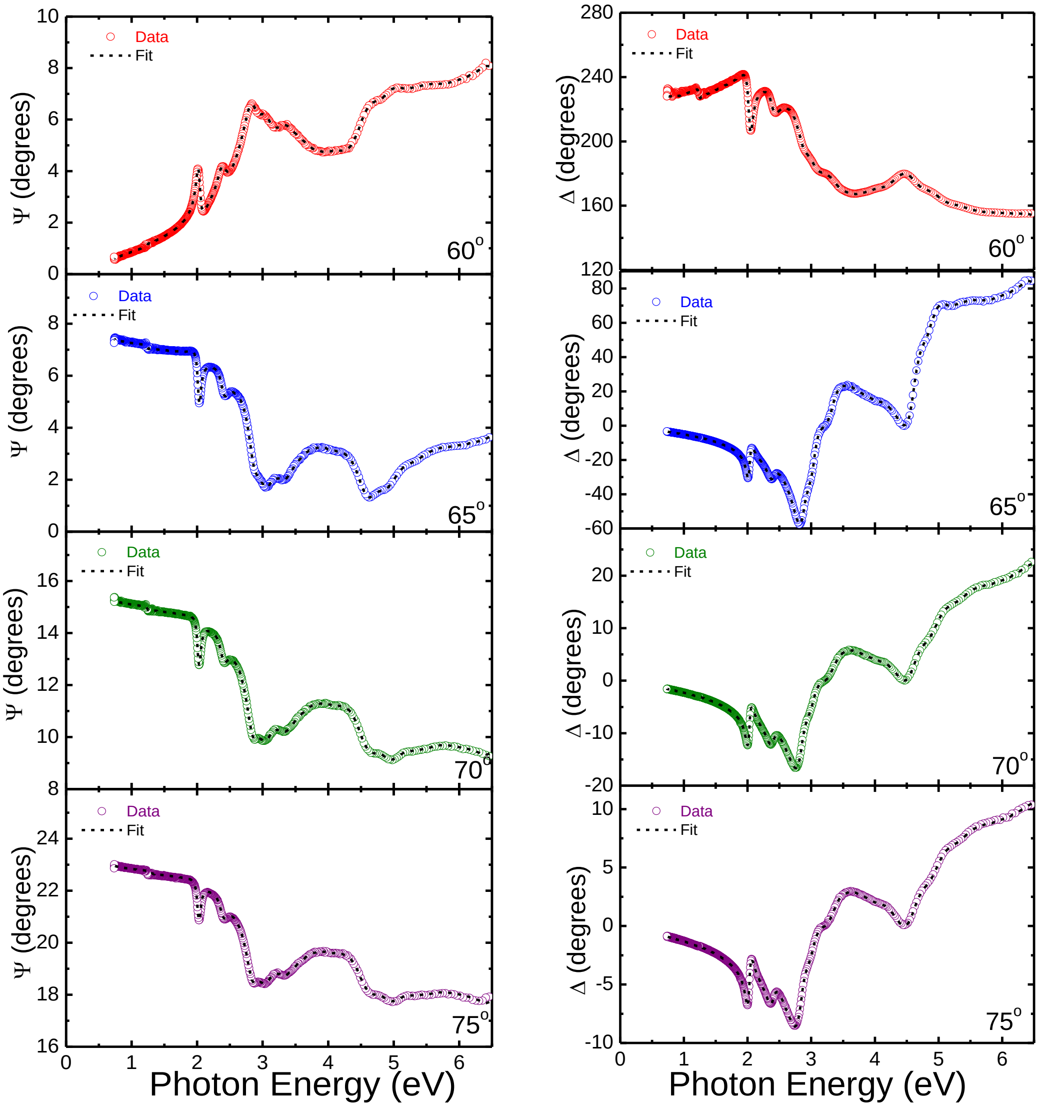

Supplementary Fig. 14 Room temperature experimental at 60°, 65°, 70°, and 75° incidence angles and fitting model of ellipsometric parameters of psi (Ψ) and delta (Δ) of monolayer WS_2.
